# The Ethics and Responsibilities of Social Media Usage by Plastic Surgeons: A Literature Review

**DOI:** 10.1007/s00266-023-03553-2

**Published:** 2023-08-17

**Authors:** Paul Oregi, Naveen Cavale, Manaf Khatib, Shakeel M. Rahman

**Affiliations:** 1 GKT School of Medical Education, King’s College London, London, UK; 2 King’s College Hospital and Guy’s and St.Thomas’ Hospitals, London, UK; 3 Lister Hospital - East and North Hertfordshire NHS Trust, Stevenage, UK; 4 Guy’s and St.Thomas’ Hospitals, London, UK

**Keywords:** Social media, Medicolegal, Ethics, Professionalism

## Abstract

**Background and Objectives:**

Social media has revolutionised how plastic surgeons advertise their work and promote their services, but concerns have been raised regarding the ethics of these practices. This review aims to identify said concerns and develop measures to address them.

**Methods:**

PubMed, Cochrane and Medline were searched for studies assessing the ethics of social media use by plastic surgeons. Five search terms were used and 23 studies identified. Results were catalogued according to which principle of medical ethics was infringed.

**Results:**

Autonomy: Patients must not be coerced into allowing their operative media to be shared and content anonymised by removing identifiable features and scrubbing metadata. Beneficence: It is difficult to balance the benefit to patients of posting photographs for educational purposes with the risk of identifiable features being present, particularly within craniofacial surgery. Non-maleficence: Taking operative media could be a distraction from the patient and lengthen the procedure which could lead to harm. Any content posted on social media should be adapted to avoid trivialisation or sexualisation. Justice: Surgeons should not entertain their audience to increase their following at the expense of patients.

**Conclusions:**

Greater oversight of social media use by plastic surgeons is required to avoid patient harm and tarnishing of the specialty’s professional standing. Professional bodies should be tasked with devising a course dedicated to the responsible use of these platforms. This should ensure the public’s trust in the specialty does not become eroded and patients are not harmed by unethical social media use.

- This review highlights the relevant shortfalls of SoMe use by plastic surgery
- Several proposals are made to reduce the incidence of these shortfalls and to ensure SoMe is used in a professional and responsible manner
- It also lists areas of the specialty where SoMe is underused and could be of help, such as academia

**Level of Evidence IV:**

This journal requires that authors assign a level of evidence to each article. For a full description of these Evidence-Based Medicine ratings, please refer to the Table of Contents or the online Instructions to Authors http://www.springer.com/00266.

## Introduction

Social media (SoMe) has brought the world closer together in an unprecedented manner, and the surgical community has been no exception to this. SoMe has enabled surgeons to reach an increasingly large audience which has not only improved patient education but has also allowed for greater collaboration initiatives between surgeons worldwide.

However, the biggest impact SoMe has had on this community has been the promotion of a surgeon’s work through these platforms, largely for commercial purposes. Plastic surgeons in particular have led the adoption of these communication tools for practice marketing and expansion, as well as delivery of educational content for both patients and trainees in the specialty [1]. Within this field, aesthetic procedures have been heavily promoted due to their consumer driven nature [2]. This trend has led to several positive changes for patients and healthcare professionals. Firstly, it has become easier for patients to access and understand relevant information regarding a procedure, such as its limitations and risks, improving the decision-making process and allowing the patient to be better informed [3]. Secondly, patients are now able to select their surgeon from a wider range of providers, as websites such as “Doctify” allow them to find most of the surgeons within a field in a particular location. Thirdly, videoconferencing platforms such as Zoom have given patients access to surgeons outside of their immediate geographical location. This is of relevance if a patient is seeking a specific procedure which is not offered by providers in their region.

However, several ethical issues have emerged around the use of SoMe by plastic surgeons. Aggressive marketing policies may end up targeting individuals on SoMe with self-esteem or body-dysmorphia problems due to the content selection algorithm used in platforms such as Facebook or Instagram [4]. These individuals may have unrealistic expectations of what can be achieved surgically [5], putting them at risk of undergoing unnecessary procedures, which are not without risks. Photographs of previous procedures may also have been edited to enhance their marketing potential, which could make already unrealistic expectations from these patients drift further away from what surgery can achieve [6]. These photographs could be accessible to individuals from certain age groups, such as teenagers or younger children, who make up a substantial portion of certain platforms’ userbases, with 21.1% of all SnapChat users being between 13 and 17 years of age as of 2022 [7]. They are at a key stage of their mental and physical development, and access to inappropriate content such as post operative photographs may alter their preconception of what their own or others’ bodies should look like. Coupled with the rise of influencers posting heavily edited content showcasing virtually unrealistic body shapes, a whole generation of young SoMe users may be at a greater risk of becoming dissatisfied with their own bodies [8]. This is a vicious cycle, as users posting heavily edited photographs are associated with greater follower numbers, which in turn may push the creator to edit their content further [9].

Lastly, a recent study determined that the vast majority of plastic surgery posts are from individuals promoting their aesthetic practice who are either doctors not trained in plastic surgery, or in some cases not even doctors at all, with board certified plastic surgeons representing only 6% of all posts [10]. Combined with the lack of awareness amongst members of the public of the board requirements for plastic surgeons [11], this could lead to patients choosing a practitioner based on their SoMe use instead of their professional credentials, with the subsequent safety consequences this could bring.

Having introduced the topic of how SoMe is used in surgery, this study will perform a review of the literature to identify how plastic surgeons use SoMe and whether this use is ethical and responsible.

## Materials and Methods

A review of the literature published between March 2000 and March 2022 was completed. The former date was chosen as it precedes the founding of the major SoMe platforms used today. Regarding the PICO framework for this review, the population assessed was the global community of plastic surgeons, whilst the intervention was the use of social media in their practices. The four guiding principles of medical ethics were used as controls, and if none were applicable to a particular shortcoming, the code of ethics of the American Society of Plastic Surgery was used (ASPS) [12]. The four pillars of medical ethics include: respect for autonomy of the patient; beneficence, doing what’s best for the patient; non-maleficence or “do no harm”; and justice, which in this context relates to balancing any competing interests between the surgeon and patient [13]. The ultimate outcomes were threefold: to identify what type of SoMe platforms plastic surgeons use, whether these vary depending on the surgeon’s type of practice, and the ethical shortcomings in how plastic surgeons currently use these platforms. Only studies in the English language were included in the review. The search was conducted in the PubMed, Cochrane and Medline databases using the following terms, which were combined appropriately: “Cosmetic Surgery”, “Social Media”, “Plastic Surgery”, “Aesthetic Surgery”, “Instagram”.

The criteria for an article to be included in the review were the following: Firstly, the articles must be a primary source of research, which excluded systematic and literature reviews. Secondly, articles that focused on SoMe use by allied specialties who also engage in aesthetic practice, such as ear, nose and throat (ENT) or maxillofacial surgery were also excluded. Thirdly, studies had to include either how surgeons used social media, including their main purposes, and/or whether this use was ethical and responsible. The data extracted from each study included the authorship, date and journal of publication, type of study, the impact factor of the journal of publication, and the main conclusions drawn from the study.

## Results

The initial search yielded 518 studies. Fifteen of these were duplicates and removed ahead of title and abstract screening. A total of 435 studies were screened, which left 63 articles to be read in full. Twenty-four studies were found to be suitable for inclusion in this review (Table 1).

**Table 1:** Summary of all papers included

Title	Author and date of publication	Journal of publication	Type of study	Impact factor of journal
#PlasticSurgery: A Comparative Deep Dive Analysis into Social Media and Plastic Surgery	[14]	Plastic and Reconstructive Surgery	Content analysis	4.73
Analysing the Quality of Aesthetic Surgery Procedure Videos on TikTok	[15]	Aesthetic Surgery Journal	Content analysis	4.28
Can Plastic Surgeons Maintain Professionalism within Social Media?	[16]	AMA Journal of Ethics	Opinion piece	1.22
Conceptualizing Professionalism in Social Media: A Framework for Evaluation	[17]	Plastic and Reconstructive Surgery	Commentary	4.73
Current Trends in the Use of Social Media by Plastic Surgeons	[18]	Plastic and Reconstructive Surgery	Survey	4.73
Framework for the Creation of Ethical and Professional Social Media Content	[19]	Plastic and Reconstructive Surgery	Guideline creation	4.73
Plastic Surgery Faces the Web: Analysis of the Popular Social Media for Plastic Surgeons	[20]	Plastic and Reconstructive Surgery Global Open	Content analysis	1.57
Insta-grated Plastic Surgery Residencies: The Rise of Social Media Use by Trainees and Responsible Guidelines for Use	[21]	Aesthetic Surgery Journal	Guideline creation	4.28
Plastic Surgery-Related Hashtag Utilization on Instagram: Implications for Education and Marketing	[22]	Aesthetic Surgery Journal	Content analysis	4.28
Social Media: A Necessary Evil?	[23]	Aesthetic Surgery Journal	Editorial	4.28
Social Media and Consent: Are Patients Adequately Informed?	[24]	Plastic and Reconstructive Surgery	Opinion piece	4.73
Social Media Guidelines for Young Plastic Surgeons and Plastic Surgery Training Program	[25]	Plastic and Reconstructive Surgery	Guideline creation	4.73
Social Media Use among Plastic and Reconstructive Surgery Residency Programs in the United States	[26]	Plastic and Reconstructive Surgery	Survey	4.73
Social Media Use and Impact on Plastic Surgery Practice	[27]	Plastic and Reconstructive Surgery	Survey	4.73
The Evolution of Patients’ and Surgeons’ Perspectives Towards the Role of the Internet and Social Media in Breast Augmentation Over 5 Years	[28]	Aesthetic Surgery Journal	Survey	4.28
The Marriage of Plastic Surgery and Social Media: A Relationship to Last a Lifetime	[29]	Aesthetic Surgery Journal	Editorial	4.28
Time for a Consensus? Considerations of Ethical Social Media Use by Pediatric Plastic Surgeons	[30]	Plastic and Reconstructive Surgery	Opinion piece	4.73
Tips and Pearls on Social Media for the Plastic Surgeon	[31]	Plastic and Reconstructive Surgery	Tutorial	4.73
Usage trends, perceptions and ethical views regarding social media: A survey of Canadian plastic surgeons	[32]	Journal of Plastic, Reconstructive and Aesthetic Surgery	Survey	2.74
When Is Advertising a Plastic Surgeon’s Individual “Brand” Unethical?	[33]	AMA Journal of Ethics	Opinion piece	1.22
When Is Posting about Patients on social media Unethical “Medutainment”?	[34]	AMA Journal of Ethics	Opinion piece	1.22
#Trending: Why Patient Identifying Information Should Be Protected on social media	[35]	Plastic and Reconstructive Surgery	Opinion piece	4.73
YouTube for Cosmetic Plastic Surgery: An Effective Patient Resource?	[36]	Aesthetic Surgery Journal	Content analysis	4.28
In Constant Search of ‘‘Like’’: How Technology and Social Media				
Influence the Perception of our Body	[37]	Aesthetic Plastic Surgery Journal	Commentary	2.7

Opinion pieces were the most common type of article (*n*=6), followed by survey-based and content analysis studies (*n*=5). The most common journal of publication was *Plastic and Reconstructive Surgery* (*n*=11), which also had the highest impact factor (*n*=4.73), Due to the heterogeneity of the studies included in the review, these were grouped according to study type and their results presented in separate tables. The editorials, commentary and tutorial were grouped with the opinion pieces (Table 2).

**Table 2:** Conclusions of opinion articles

Title	Main conclusions
Can Plastic Surgeons Maintain Professionalism within Social Media?	The authority of the ASPS code of ethics is limited by restraint of trade regulations. It is key to ensure there is no coercion from surgeons to make patients take part in marketing. It is recommended to use independent videographers so surgeons focus on the tasks at hand. Identifying features should be removed and metadata scrubbed. Patient should be informed that images may not be removable in future. Surgeons ought to ensure that websites show real people with real results and should state where models have been used.
Social Media and Consent: Are Patients Adequately Informed?	Posting medical images on social media is different to academics as a surgeon is not protected by copyright and may lose control of them. Professionals should advocate for an additional level of consent known as “Use of patient information and/or photographs on social media” to ensure patients are aware their photographs may never be fully removed from the internet.
Time for a Consensus? Considerations of Ethical Social Media Use by Paediatric Plastic Surgeons	Maintaining privacy and obtaining consent are the most critical issues. This becomes nearly impossible in craniofacial surgery. As well as consent from guardians, assent is also important, which is the agreement of a participant who legally cannot consent. This is not always possible in toddlers, so surgeons must weigh up the benefits and ensure content is beneficial for the patient and not purely for personal gratification purposes.
When Is Advertising a Plastic Surgeon’s Individual “Brand” Unethical?	Deception in social media advertising is the first pitfall, carried out by removal of negative reviews or by suggesting a treatment is exclusive and scarce. This can lead to unrealistic expectations and may result in disappointment and a sense of betrayal. As the relationship of trust has been built on social media and may not be as close, this sense of betrayal may lead to withdrawing from follow-up care or self-recrimination.
When Is Posting about Patients on social media Unethical “Medutainment”?	Using patient images requires adherence to the Health Insurance Portability and Accountability Act (HIPAA). Plastic Surgeons should maintain separate personal and professional accounts and minimise online interactions with patients. Patient confidentiality must be maintained and images de-identified. Surgeons also ought to show patients their images or videos before posting them. Context is key and media content needs to be adapted to avoid sexualisation. Reassuring patients that their care will be unaffected if they refuse to share their media is also vital.
#Trending: Why Patient Identifying Information Should Be Protected on social media	Three recommendations: Firstly, all images need to be censored for anonymity, including “identifiable facial features, tattoos and other unique cutaneous features”. Secondly, inform patients that images can be “saved, manipulated and downloaded” even after deletion. Thirdly, encourage internal governance to avoid harm to patients, surgeons and the field of plastic surgery
Social Media: A Necessary Evil?	Beneficence: It is not always easy to see how a patient benefits from a surgeon posting their images online. Non-maleficence: Generally, patients are at a higher risk of being harmed than surgeons from social media, as it is impossible to know if a post might go viral and reveal their identity. Surgeons need to be transparent and obvious if they are being paid and sponsored for a post. There is also a concern that the doctor-patient relationship is being distorted. Removing engagement metrics, such as followers or likes could reduce deceit from surgeons using these metrics. Residents should not post surgical results as their own, as ultimately the patient’s responsibility lies with the attending surgeon. There need to be clear distinctions between social media and a surgeon’s own website.
The Marriage of Plastic Surgery and Social Media: A Relationship to Last a Lifetime	Plastic Surgeons should aim to handle negative reviews in a constructive manner, which can help demonstrate knowledge, compassion and attention to patient issues. Consulting with professional societies and an attorney to ensure posts are of a high ethical and legal standard is highly encouraged
Tips and Pearls on Social Media for the Plastic Surgeon	Patient safety: Patient information needs to be deidentified and social media should not be allowed to affect care (anaesthetic lengthening to take photographs). Truthfulness: There needs to be transparency in all shared information and permission should be obtained with the new social media specific consent form once it is released Professionalism: Aim to keep personal and professional accounts separate and avoid inappropriate/unprofessional content. Thoughtfulness: Surgeons need to remember that as ambassadors on these platforms for their specialty, they ought to consider how viewers may react to certain content (gore, body parts). Duty: Plastic Surgeons should always dissociate from individuals not upholding professional standards as well as report violations to the relevant professional bodies.
Conceptualizing Professionalism in social media: A Framework for Evaluation	There are four points to determine the professionalism of a social media post: Context: Ensure a post does not trivialise patient experience. Intent: Educating the public or promotion of one’s practice both have professional intentions, but anything related to self-promotion should be kept on a separate personal account. Content: Dialogue should include professional language. Any form of sensationalism or indifference to surgical specimens are to be avoided. The pose of a physician should be similar to when taking a professional picture. Presentation: No emojis or filters should be used, as these can trivialise the trust of a patient towards their physician and are unprofessional. Hashtags need to be in the style of a journal figure legend and use professional language.
In Constant Search of ‘‘Like’’: How Technology and Social Media Influence the Perception of our Body	The increased use of SoMe during the pandemic led to individuals becoming hyperaware of facial imperfections which had previously gone unnoticed..These can be caused by a number of technical factors, and Plastic Surgeons should raise awareness of how they cause these distortions.

## Opinion Articles

*Autonomy* Most studies agreed that an additional level of consent was required when asking patients for permission to upload their operative media onto SoMe. This consent form had to ensure patients were aware that once uploaded, these photographs may no longer be fully deletable, and may even cease to be the property of the surgeon. It was also encouraged that surgeons show their patients the media before it is uploaded to aid the process of informed consent. Patients must not be coerced into consenting and the surgeon should stress that care would not be affected by this decision. Any content uploaded onto SoMe must be fully anonymised, which should be carried out by removing identifiable characteristics and scrubbing metadata.

*Beneficence* Due to the nature of craniofacial surgery, one study highlighted the difficulty of balancing the benefits of social media to this patient population with the higher risk of confidentiality breach associated with facial photographs and videos.

*Non-maleficence* A study mentioned that a separate videographer should be required to avoid distracting the surgeon from the patient and lengthening the anaesthetic time. Any content posted should be adapted to ensure it does not sexualise or trivialise the patient’s experience.

*ASPS code of ethics* Surgeons were encouraged to have separate personal and professional accounts and keep their interactions with patients via their personal account to the minimum. Surgeons also ought to state where models have been used, only show real results, and avoid photograph editing (Table 2).

**Table 3:** Conclusions of survey based studies

Title	Main conclusions
Current Trends in the Use of Social Media by Plastic Surgeons	Salaried users used social media for non-medical interests (75%), public education (38.3%) and networking (35%). Private practice users were similar for public education, but also used them for patient acquisition (74.3%) and practice branding (61%). Both groups had Facebook, but only private practice used Instagram and Pinterest. Salaried non-users were more concerned about breach of patient privacy (60% vs 38.9%). Compared to non-users, more social media users thought before and after photographs and reviews were acceptable. Within this, younger, aesthetic focused surgeons were more likely to find these acceptable.
Social Media Use among Plastic and Reconstructive Surgery Residency Programs in the United States	85% of residency programs using social media had Instagram. Most common uses were branding (77%), education (74%) and residency recruitment (66%). Only 69% of faculties had guidelines for SoMe use, 51% of programs had no training for residents and faculty, and only 58% of programs had reprimands in place for inappropriate use. Breach of patient privacy was found in 11% of programs and 31% had professionalism concerns.
Social Media Use and Impact on Plastic Surgery Practice	Most respondents had a largely cosmetic practice (32%). Nearly equal numbers were users vs non users (50.4% and 49.6%, respectively). Facebook was the most used social media for all surgeons. 75.1% of users had separate personal and professional accounts. Top reasons for use included “incorporation of social media into practice is inevitable” (56.7%), “effective marketing tool” (52.1%) and “provides a platform for patient education” (49%). Reasons for not using social media included concerns about maintaining professionalism (54.1%), preserving patient confidentiality (48.8%) and becoming too accessible (45.9%). 24.5% felt governing bodies should have more oversight of plastic surgeons’ use of social media.
The Evolution of Patients’ and Surgeons’ Perspectives Towards the Role of the Internet and Social Media in Breast Augmentation Over 5 Years	% of surgeons who thought the internet and social media led to better information declined over time (61.7% in 2014, 42.0% in 2017 and 35.4% in 2019). Similar thing occurred with social media leading to unrealistic expectations (38.3% in 2014, 56.5% in 2017 and 65.3% in 2019). However, number of respondents who thought plastic surgery content should be removed from social media went down (21.9% in 2014 to 9.7% in 2019). 97.9% of respondents in 2019 had a presence on social media and 67% posted content related to surgery and post-operative results. Top 5 platforms were Facebook (74.7%), Instagram (64.2%), LinkedIn (28.4%), Twitter (17.9%) and Snapchat (5.3%).
Usage trends, perceptions and ethical views regarding social media: A survey of Canadian plastic surgeons	37% had a single account for personal and professional use, 27% just personal, 19% just professional, only 5% had two accounts. Those with just a professional account had the most active usage (posting at least once a day). 45% thought it was not ethical to discuss procedures with patients over social media. Surgeons without a professional account more likely to believe posting operative photographs is unethical (75% vs 21%). Surgeons without a professional account were also more likely to have an academic portion to their practice (100%) compared to those with a professional account (68%).

## Survey Studies

Most studies agreed that amongst all board-certified plastic surgeons, Facebook was the most popular platform. Instagram was also a popular option but was used more extensively by those with a cosmetic practice and residents. Both academic and private practice surgeons used SoMe in similar proportions when it came to education and networking. However, academic surgeons barely used these platforms for promoting themselves, unlike private practice providers. Younger, aesthetic providers who used SoMe were much more likely to view posting before and after photos, patient testimonials and reviews as ethically acceptable compared to older, reconstructive surgeons who did not use SoMe.

Most residency programs using SoMe had Instagram as their preferred platform. Only half of them provided formal training to staff on how to use these platforms, and 11% reported breaches in patient confidentiality, although it was not stated whether this was due to inappropriate SoMe use. The main reasons for non-use of these platforms included too time-consuming, concerns regarding breaching of patient confidentiality, and maintaining professionalism within the specialty. American surgeons were more likely to maintain separate personal and professional SoMe accounts than Canadian ones. A large majority of Swedish surgeons maintained a presence on SoMe and regularly posted operative content on these platforms. This was despite reporting increasingly negative views on the quality of plastic surgery information available online, as well as believing that SoMe had made patients’ expectations increasingly unrealistic (Table 3).

**Table 4:** Conclusions of content analysis studies

Title	Main conclusions
Plastic Surgery-Related Hashtag Utilization on Instagram: Implications for Education and Marketing	Hashtags using lay terminology were more likely to have more posts associated with them than those with medical terminology. Board certified surgeons only accounted for 17.8% of top posts, other specialties marketed themselves as “cosmetic surgeons”. Board certified plastic surgeons were more likely to post educational content as opposed to self-promotional content.
YouTube for Cosmetic Plastic Surgery: An Effective Patient Resource?	Only 37% of videos included a board-certified surgeon. These had a higher DISCERN score and lower bias than those without these providers. However, they also attracted a lower number of viewers. Videos by academic institutions had the highest DISCERN score but also represented a small proportion of the total content.
Analysing the Quality of Aesthetic Surgery Procedure Videos on TikTok	DISCERN score were very poor for all four procedures (1.25-1.55). Higher scores were found for educational videos and those posted by doctors.
#PlasticSurgery: A Comparative Deep Dive Analysis into social media and Plastic Surgery	Plastic surgeons and clinics represented 54% and 61% of posts on Twitter and Instagram, respectively. Tweets were predominantly educational and focused on basic science and patient safety (38.6 vs 9% on Instagram). Instagram content was more likely to be promotional or outcome based and contain intraoperative photographs or videos. Identifiable patient features were more common on Instagram (13.4% vs 1.6% on Twitter); these posts also had higher user engagement and became trending posts (19.5%). Post engagement was overall much higher on Instagram.
Plastic Surgery Faces the Web: Analysis of the Popular social media for Plastic Surgeons	63% of posts on Instagram were by surgeons compared to 18% on Facebook and 13% on YouTube. Educational posts represented only 16% of total. Images of women were also much more common in advertising (68%). Educational posts were much less successful in attracting attention. Posts involving shaming also attracted more attention. Videos of surgeries, in some case provocative, also attracted more likes, as well as ‘attractive’ female plastic surgeons.

## Content Analysis Studies

*Autonomy* High rates (13%) of patient identifying features were found on Instagram. These posts also had higher user engagement and were more likely to become trending.

*Beneficence* Most educational, high-quality posts on TikTok and YouTube considered beneficial for patients were posted by board certified plastic surgeons. Educational videos also had lower engagement rates and were less common than non-educational, lower quality videos. Twitter was used predominantly for education on patient safety and clinical science, and most plastic surgery-related posts were uploaded by board-certified plastic surgeons. However, post engagement on Twitter was significantly lower than on Instagram

*Justice* A large proportion of Instagram content was promotional and contained operative media. Given that a substantial number of these posts were found to breach patient confidentiality, this could tilt the competing interests of surgeon and patient towards the former, at the expense of the latter’s information being leaked (Table 4).

**Table 5:** Conclusions of guideline creation articles

Title	Main conclusions
Framework for the Creation of Ethical and Professional Social Media Content	Need to disclose if purchasing followers, otherwise could be considered false advertising. Providing free/discounted services to patients in exchange for publicity is a violation of multiple ethical codes, and needs to be disclosed. Surgeons should be fully familiar with each social media platform’s T&Cs. Some companies also take ownership of photographs from the user after being posted, therefore surgeons need to familiarise patients with this. Physicians advised against reposting, retweeting or responding to posts as they are at risk of violating HIPAA if the posts breached confidentiality.
- Insta-grated Plastic Surgery Residencies: The Rise of Social Media Use by Trainees and Responsible Guidelines for Use	- Surgeons should: - Avoid engaging in controversial accounts. - Avoid using live streaming as you cannot control live content. - Avoid unprofessional hashtag use. - Censor or delete material from other users that could be considered inappropriate and damage the poster’s brand. - Avoid posting identifiable information, including what type of surgery and where, as this could identify the patient. - Avoid content where surgeon is performing any action other than clinical duties, such as dancing or posing. - Avoid blood and gore, as posts may be accessible by the general public. - Consider a course on social media for residents.
Social Media Guidelines for Young Plastic Surgeons and Plastic Surgery Training Program	Surgeons must: Be clear that patients may refuse involvement with social media with no effect on care. Inform patients that once posted, photographs may become property of the platform and can never be fully removed. Use a dedicated videographer to avoid lengthening anaesthesia. Use professional hashtags when engaging in educational activities online. Avoid photographic editing and state length of time between before and after Avoid establishing a patient-doctor relationship online, do not provide medical advice. Not entertain an audience at the expense of a patient. Not respond to negative reviews on rating websites or ask friends and family to write positive ones. Fully disclose any paid sponsorships on social media posts. Promotion of plastic surgery related causes is allowed as long as it is done “tastefully and respectfully”. Residents cannot present their attending’s work on social media as their own, not only need patient’s permission but also their attending’s.

## Guideline Creation Studies

*Autonomy* One author suggested surgeons should be aware of SoMe platforms’ T&Cs and ensure patients were too. If these were to change too often, the surgeon should state this to the patient and explain the T&Cs may change in the future. Surgeons were also advised against reposting or retweeting other physicians’ work, as this could incur in a HIPAA violation should the photograph fall foul of this regulation. Live streaming was also advised against as it cannot easily be controlled and may lead to confidentiality breaches.

*Non-maleficence* Surgeons should not perform any non-clinical activities which trivialise the patient experience. Blood and gore also ought to be avoided, as they have the potential to cause distress to other users. Surgeons were advised to not establish a doctor-patient relationship online as this could lead the patient to develop a false sense of trust towards the physician.

*Justice* Surgeons should not entertain their audience to increase their fame at the expense of a patient.

*ASPS Code of ethics* Purchasing followers, unprofessional hashtag use by using lay terms instead of the appropriate medical equivalent; and not disclosing any paid sponsorships would all break this code. Surgeons were also encouraged to state the length of time in before-and-after posts so patients were adequately informed, preventing unrealistic expectations (Table 5).

## Discussion

Since its inception, SoMe has become increasingly linked with the field of plastic surgery. Even residency programs have embraced apps like Instagram for education and recruitment of prospective students. However, the uptake of SoMe by academic plastic surgeons and journals has lagged behind other specialties. These journals do not easily allow users to share links to content via their own accounts [38], and one study even found that plastic surgery journals only posted 3% of tweets containing the word “plastic surgery”, and none contained a link to a journal or a reviewed article [39]. Given the role of journals in the education of future surgeons and their patient population, it is imperative they increase their presence and reach on SoMe. This would improve the quality of plastic surgery information on these platforms and reduce misinformation.

Twitter in particular has been found to be useful when helping journals improve their impact factor. One study in particular found that 5 of 6 journals which joined Twitter increased their impact factor since joining this platform [37]. It has even been shown that using SoMe to spread research is associated with higher citation rates [40]. Given the educational potential that this platform has, all plastic surgery journals should establish a presence to broaden their reach and boost their impact.

Plastic surgeons have also been found to be responsible for very little of all plastic surgery content on streaming platforms such as Youtube. Most of the content on this platform has very little educational content, is highly biased and tends to focus on the aesthetic benefits of procedures rather than the risks involved. This was confirmed by a systematic review on the use of YouTube for educational purposes, which found it to be generally lacking [41]  Given that patients use these platforms to gain medical knowledge, this could greatly reduce their risk perception of aesthetic procedures, with the harm this could bring. An effort should also be made by board-certified individuals to increase the quality and quantity of educational videos on live-streaming platforms.

Furthermore, given the young audience that uses these apps, there is a risk that individuals who are at a crucial stage of development could access wholly inappropriate videos with long lasting psychological effects. Therefore, surgeons should aim to prevent these users from accessing any explicit material by placing age-related restrictions. If a platform does not allow these, surgeons should refrain altogether from posting this type of content on that platform.

However, the platform which has seen the most usage by plastic surgeons is Instagram. It is mostly used for promotional purposes and is the domain of private practice surgeons due to its emphasis on visuals. However, an alarmingly high number of posts breaching patient confidentiality have been found on this platform [14]. What’s more, these posts also have higher engagement rates and are more likely to go viral, further spreading the patient’s identity. This is not only a legal problem but also a breach of the fundamental code of ethics by which all surgeons should abide. Therefore, if surgeons want to use patient media for commercial purposes, a separate consent process should be carried out. The ASPS website contains a proposed consent form this purpose, which should be instituted and required for all plastic surgeons to use [42]. This form could be improved by incorporating patient groups into the process, allowing aspects of the consent process that surgeons may not have thought about to be incorporated. The form would then be adapted by surgeons from other countries to ensure it is compliant each legal framework.

It is also imperative that filters and photograph alterations are always avoided. Not only would this fall foul of ethical codes, but in certain jurisdictions this could also be considered false advertising [16]. Filters should also be avoided to recreate what the post-operative result of a procedure may look like, particularly with facial operations such as Rhinoplasty, as these filters cannot accurately predict the result of a procedure and may alter the patient’s expectations.

The rise of these filters and the popularity of the “selfie” on Instagram have caused what some plastic surgeons call a “selfie pandemic” [43]. These beauty filters alter the appearance of the skin to make it look smoother. This appearance is simply impossible to replicate with any form of aesthetic treatment, as it represents an ideal which is not attainable with real human tissue that is constantly changing in response to the environment it is in [37]. A similar phenomenon has occurred during the pandemic with the increased use of remote working apps such as Zoom, which has been coined “Zoom dysmorphia”, and has made people hyperaware of facial features they see as “imperfect” [44] . These imperfections are often due to technical factors related to the camera or the light settings, such as distortion due to the focal length of a lens, or inappropriate lighting that leads to a light-shadow reflex causing an imperfection which is not really present. It is thought that these two phenomena have caused a great increase in the number of patients seeking to undergo facial plastic surgery to improve their “selfie” look, particularly the periocular region. There are several theories why this is the case, one of them being that the eyes were often the only part of the face that is visible when wearing a mask, or that they are the first aspect of someone’s face that others notice on photographs or video [37]. Plastic surgeons need to be aware of this phenomenon, as these patients may often have unrealistic expectations of what can be achieved from surgical interventions, which will simply not be able to replicate or live up to the expectations generated by filters and the search for a “perfect selfie”. These patients should be clearly distinguished from those were there is an objective anatomical feature which can be feasibly surgically modified into a desirable shape or size. Surgeons ought to counsel patients on the factors listed above in order to avoid disappointment, which could even turn into frustration and resentment should the surgeon’s promise to achieve an unattainable result go unfulfilled. This could have significant repercussions for the doctor-patient relationship, and potentially even long lasting psychological or physical sequelae for the patient, as no operation is without risk.

Even though it has been recommended in the literature to use livestream features such as “Instagram Live” to increase user engagement [31], in the view of these authors they should be avoided. This is because the content being uploaded cannot be controlled in the same way that previously taken photographs and videos can. It cannot be edited and adapted to make it suitable for social media use, and due to the unpredictable nature of livestreaming there is also an increased risk of confidentiality breach [21]. Livestreaming may also deviate the surgeon’s attention from the patient to the camera to focus on entertaining users, with the dangers this could bring. This can occur even when just taking photographs and videos and may result in lengthening of the procedure for the purpose of photo-taking. Therefore, an independent videographer should always be used for recording media. It is currently unknown how many surgeons use videographers for operative media.

Lastly, surgeons should always aim to keep separate personal and professional social media profiles and keep all self-promotion on their personal account. Contact with patients on personal accounts must be avoided and even when contacted on a professional account, surgeons should refrain from giving any form of medical advice on SoMe. This is done to prevent patients from building a relationship with their physician on SoMe platforms, which could lead the patient to form a false sense of trust with their doctor and even view them as a friend [23]. This would heavily distort the doctor-patient relationship and could lead patients to feel a sense of betrayal towards their surgeon should they be disappointed with the result of a procedure.

To improve on the shortfalls listed above, we believe that altering guidelines of use or changing a society’s code of ethics would not be sufficient. Instead, professional associations should develop with a SoMe-specific course which surgeons must complete to continue using these platforms. It should include descriptions of all the shortcomings listed above, how to avoid them, and general tips on what is considered best practice when using SoMe for any purpose. Patient representative associations could also be asked to contribute to ensure the course is devised with inputs from all relevant players. The course’s success would then be measured by carrying out surveys to check if surgeons have incorporated the practices learnt, or if certain individuals continue to engage in non-ethical practices. Other ways to measure this course’s success could be to assess plastic surgery content after implementation to check if the quality and quantity of educational content had improved, or if the number of Instagram posts containing confidential features had reduced in number, to list a few outcomes.

These surveys could also ask for points of improvement, which would then be incorporated into future iterations of the course. A public health campaign could also be carried out to educate patients on the main pitfalls of SoMe use by plastic surgeons, and how to identify and avoid providers who fall foul of these. The British Association of Aesthetic Plastic Surgeons (BAAPS) has carried out a similar campaign known as “Do your homework”, which encourages patients to check if a provider is on the UK specialist register and has completed surgical training in the UK [45].

However, these shortcomings should not cause plastic surgeons to withdraw SoMe altogether, as this could also put patients at risk in several ways. Firstly, it would remove most credible sources of plastic surgery education for patients from online media, at a time when more patients than ever are obtaining healthcare information from online sources. Secondly, it would mean non-certified providers would be able to market their services on SoMe platforms with no board-certified practitioners to be compared against. These non-certified providers may practice in jurisdictions with looser professional regulations and provide their services at a lower cost, resulting in cosmetic surgery patients travelling abroad to undergo a procedure, a practice known as cosmetic tourism. This often ends in disaster, with patients not receiving proper information on the risks of a procedure and being unable to contact their surgeon should complications occur. These patients may end up requiring costly emergency treatment in their home country, with life-long physical and mental consequences [46] .

There have been several scenarios where collaboration via SoMe has resulted in a beneficial outcome for both surgeons and patients. A prime example of this is the International Microsurgery Club, a global collaborative platform based on Facebook. It has allowed over 8000 surgeons worldwide (as of 2019) to engage in case discussion [47].

Nevertheless, this study is not without limitations. As with any literature review, its strength is determined by the evidence available. The number of papers quantitatively assessing the behaviour of plastic surgeons on SoMe is limited, and most papers in this review were opinion-based. Furthermore, given that none of the articles from the review were randomised controlled trials, the study type offering the lowest degree of bias, it is likely the bias from the papers included is high.

This review also didn’t include studies assessing the use of instant messaging platforms such as WhatsApp. When used to spread files which may contain confidential information, such as clinical photographs or imaging, users may be falling foul of personal data regulations such as GDPR due to its use of data centres outside of the user’s country. Therefore, to avoid the ethical and legal consequences of this breach, alternative platforms that do not fall foul of data protection regulations, such as Siilo in Europe, or OhMD in the USA, should be used.

## Conclusion

Social media has changed how plastic surgeons educate patients and trainees as well as how they advertise their work. It has allowed knowledge to be transmitted beyond borders and helped trainees continue with their learning during a global pandemic. Conversely, it has also given rise to ethical problems which could jeopardise the professional standing of the specialty. Ultimately, given that SoMe will continue to grow and expand regardless of whether plastic surgeons use it or not, it is imperative that this profession continues to expand the usage of these platforms as part of their practice. This will ensure that patients continue to have reliable sources of information within this field, as well as enable them to compare and contrast professional, certified practitioners from others who lack these qualifications. This will help patients make sensible and safer choices regarding their aesthetic or reconstructive care provider. Nevertheless, it is also imperative that plastic surgeons adhere to strict ethical and legal frameworks, as summarised in the following table. This will help maintain the professional integrity of the specialty, whilst preventing patients from being harmed by unprofessional social media use or dangerous, non-licensed providers (Table 6).

**Table 6:** Main guidelines to follow when using SoMe

Pitfall	Guideline
Lack of informed consent around operative media	Aim to use SoMe specific consent form, and provide counselling around the potential loss of ownership of media once it is online
Leakage of confidential information	Scrub metadata from media and ensure content is fully anonymised
Respect of patient autonomy	Ensure care is not altered by decision of whether to share media or not
Increase of operative risk due to media taking	Use a separate videographer to avoid any distractions or lengthening of the procedure
False advertising and usage of personal accounts	Avoid use of filters or photograph editing in marketing and contact patients using a professional, and not a person account
Lack of training using SoMe	Once released, surgeons should ensure they attend any SoMe training courses devised by professional bodies such as ASPS or BAPRAS to ensure they are compliant with the latest ethical and legal standards
